# Aptamers as Theranostic Agents: Modifications, Serum Stability and Functionalisation

**DOI:** 10.3390/s131013624

**Published:** 2013-10-10

**Authors:** Sarah Shigdar, Joanna Macdonald, Michael O'Connor, Tao Wang, Dongxi Xiang, Hadi Al.Shamaileh, Liang Qiao, Ming Wei, Shu-Feng Zhou, Yimin Zhu, Lingxue Kong, Santanu Bhattacharya, ChunGuang Li, Wei Duan

**Affiliations:** 1 School of Medicine, Deakin University, Pigdons Road, Waurn Ponds, Victoria 3217, Australia; E-Mails: jmmacd@deakin.edu.au (J.M.); mloc@deakin.edu.au (M.O.); wat@deakin.edu.au (T.W.); dxiang@deakin.edu.au (D.X.); halshama@deakin.edu.au (H.A.); 2 Storr Liver Unit, at the Westmead Millennium Institute, The University of Sydney at the Westmead Hospital, Westmead NSW 2145, Australia; E-Mail: liang.qiao@sydney.edu.au; 3 School of Medical Science and Griffith Health Institute, Griffith University, Gold Coast Campus, Southport 4222, Australia; E-Mail: m.wei@griffith.edu.au; 4 Department of Pharmaceutical Sciences, College of Pharmacy, University of South Florida, Tampa, FL 33612, USA; E-Mail: szhou@health.usf.edu; 5 Suzhou Key Laboratory of Nanobiomedicine, Division of Nanobiomedicine, Suzhou Institute of Nano-Tech and Nano-Bionics, Chinese Academy of Sciences, Suzhou, Jiangsu 215123, China; E-Mail: ymzhu2008@sinano.ac.cn; 6 Institute for Frontier Materials, Deakin University, Waurn Ponds, Victoria 3217, Australia; E-Mail: lingxue.kong@deakin.edu.au; 7 Department of Organic Chemistry, Indian Institute of Science, Bangalore 560 012, India; E-Mail: sb@orgchem.iisc.ernet.in; 8 Centre for Complimentary Medicine Research, National Institute of Complementary Medicine, University of Western Sydney, Campbelltown Campus, Penrith, NSW 2751, Australia; E-Mail: chun.guang.li@rmit.edu.au

**Keywords:** aptamer, SELEX, cancer, diagnostics, locked nucleic acid, unlocked nucleic acid

## Abstract

Aptamers, and the selection process known as Systematic Evolution of Ligands by Exponential Enrichment (SELEX) used to generate them, were first described more than twenty years ago. Since then, there have been numerous modifications to the selection procedures. This review discusses the use of modified bases as a means of enhancing serum stability and producing effective therapeutic tools, as well as functionalising these nucleic acids to be used as potential diagnostic agents.

## 1. Introduction

Aptamers are simple short strands of DNA or RNA that have been generated in the laboratory using *in vitro* selection processes. In 1990 three groups concurrently described the process of the selection of nucleic acids. Ellington and colleagues generated RNA molecules capable of binding organic dyes, while Robertson and Joyce described the generation of RNA enzymes that could specifically cleave DNA, and Tuerk and Gold generated RNA ligands to a bacteriophage T4 DNA polymerase [1–3]. This ingenious process stimulated the imagination of a group of researchers who began to explore the applications of this new class of nucleic acids, with exquisite three-dimensional structures, in biology and medicine. However, using nucleic acids as therapeutic molecules was not a new idea in 1990. Antisense oligonucleotides (ASOs) were first described as a means of gene silencing in 1977 [4,5], though these ASOs have yet to realise their therapeutic potential. Whereas ASOs bind in a Watson-Crick base pairing manner, aptamers interact with their targets through the complex three dimensional shapes that these nucleic acids fold into and thus bind via an “induced fit” mechanism. For this reason, aptamers are also commonly referred to as chemical antibodies.

**Figure 1.**
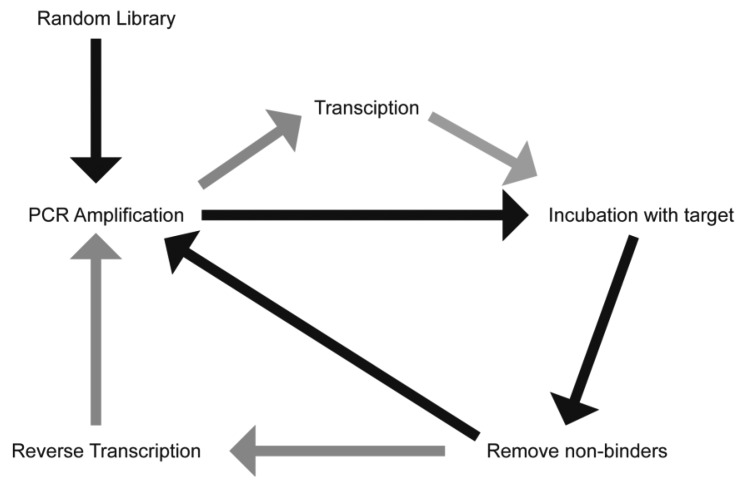
Schematic of SELEX for DNA (black arrows) or RNA (grey arrows) selection. An initial pool of randomised DNA is amplified by PCR. This pool can be transcribed into RNA prior to incubation with the target of choice. Non-binding species are removed and the binding species are reverse transcribed (RNA aptamers) prior to PCR amplification. This is repeated 8–12 times to produce an enriched pool of binding species that are highly specific for the target. Modified from [18].

The process of aptamer generation is very different from that involved in ASOs and RNAi, where knowledge of the gene sequence is required. Aptamer selection requires no prior knowledge of the target sequence or even, in some cases, what the specific target is. Aptamers have been generated against proteins, cells and even whole organisms [6–10]. There have also been recent reports describing the *in vivo* selection of aptamers using an animal model of intrahepatic colorectal cancer [11], and of aptamers capable of penetrating into the brain [12]. This selection process has been termed the Systematic Evolution of Ligands by EXponential Enrichment (SELEX) and involves iterative rounds of incubation of a randomised library with a “target” (Figure 1). While this process can be time-consuming and tedious, it does produce aptamers with a high affinity and selectivity to the target. This process can generate aptamers of either RNA or DNA, with the final application generally guiding the researcher as to the species to use. RNA aptamers are generally considered to form much more complex three dimensional shapes compared to DNA aptamers. However, unmodified RNA has a half-life of seconds in human serum, a limiting factor in generating aptamers for therapeutic applications. Both DNA and RNA species are inherently susceptible to nucleases present in human serum, though DNA aptamers generally have a half-life of approximately 60 min [13].

A key factor in developing oligonucleotides for therapeutic applications has been the inclusion of structurally modified nucleotides into the sequence. These modified nucleotides are nuclease resistance and thus resist exo- and endo-nuclease degradation. This has generally been performed following selection processes and can affect the binding properties of aptamer. It is not surprising considering aptamers possess a binding region that interacts with the target via hydrogen bonding and van der Waals interactions [14]. Through these interactions, the aptamer binds tightly through conformation-based interactions. Any structural change that occurs within the binding region of the aptamer will therefore affect the tightness of the binding, thus reducing the binding affinity or affecting the specificity of the interaction [15]. The post-SELEX modification was therefore very much a trial and error process prior to the development of computer simulation programs, such as RNAFold and CENTROIDFOLD [16,17], which can predict the two-dimensional structure of the aptamer and allow researchers to use rational design to insert modified bases into regions of the aptamer that were unlikely to interact with the target, although the binding of the modified aptamer still needs to be confirmed by experiments. A number of modified bases can be inserted into the aptamer structure in this way, thus providing a way to stabilise the aptamer for *in vivo* therapeutic applications.

## 2. Post-SELEX Structure Modification

Nuclease degradation is a major issue for therapeutic nucleic acids. Studies with ASOs provided a useful strategy to augment the nucleic acid strand in order to prevent degradation. One common method has been to cap the 3′- and/or 5′- ends of the nucleic acid strand [19], which prevents attacks by exonucleases. Endonucleases, however, appear to have a preference for pyrimidines, with cleavage occurring more frequently in the case of two or more adjacent pyrimidines [20,21]. One of the first types of modification introduced to enhance the stability of ASOs was the use of phosphorothioate nucleotides as an alternative backbone. In fact, modifying the phosphate linkage has been used fairly effectively to improve enzymatic stability. In the case of the phosphorothioate base, one of the non-bridging phosphate oxygens in the phosphodiester linkage is substituted for sulphur [22]. This has proven to be very effective in protecting ASOs against exonucleases when placed in the 5′- or 3′- end of the oligonucleotide, with it appearing to be most effective when placed at the 3′-side of the internal pyrimidine [20]. However, it has been suggested that these are “stickier” towards proteins than the normal phosphodiester backbone [23], which may present a problem with aptamers and non-specific binding.

**Figure 2.**
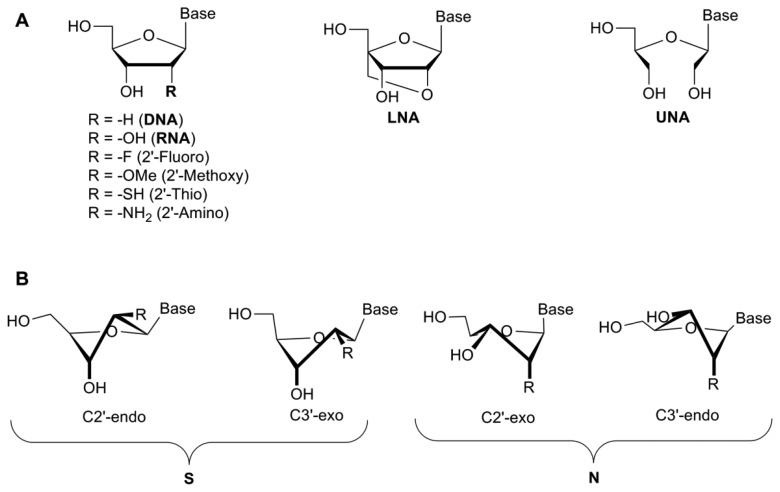
Representative structures of modified bases. (**A**): RNA bases can be modified at the 2′-OH (R) group with the substitution of fluoro, methoxy, thio or amino groups; The structure of locked nucleic acids is locked in place through a methylene bridge; The structure of unlocked nucleic acids is flexible due to the loss of the C2′-C3′ bond; (**B**): The sugar ring forms an “S” or “N” type conformation, depending on the functional group (R). Modified from [27,28].

The 2′-hydroxyl group plays a key role in aptamer folding/structure with the 2′-OH influencing the sugar conformation, though reports are conflicting as to whether this particular group plays a role in binding to the protein through specific hydrogen bonding interactions [24–26]. Modifications of the 2′-position of the ribose sugar have been shown to aid in stronger base-pairing with the target, which can lead to increased binding specificity and RNase/nuclease resistance [19] by removing the 2′-hydroxyl group from the sugar which is the naturally occurring cleavage site prone to nucleophilic attack [10]. These modifications include the incorporation of amino (NH_2_), fluoro (F), alkyl and thio groups to the 2′-position (Figure 2A).

A study demonstrated that these types of modification increased serum stability from less than one second for a completely unmodified RNA aptamer, to more than 81 h [10]. Interestingly, modification of only one of the pyrimidines (cytosine or uridine) did not increase the half-life significantly (≤1 min) [10]. However, the modification of all pyrimidines with 2′-fluoro-pyrimidines resulted in a significant increase (81 h). When mixed bases were introduced into the structure of the aptamer (2′-F-C & 2′-NH_2_-U or 2′-F-U & 2′-NH_2_-C), the half-life increased to 36 h. The incorporation of 2′-aminopyrimidines into all C and U positions, however, increased the half-life to approximately 367 h. While the serum stability was significantly increased, the aptamer lost all binding capacity following the incorporation of 2′-NH_2_-pyrimidines [10] (Table 1). This may be because the 2′-amino groups favour a C2′-endo pucker, similar to the DNA β-helix, while 2′-fluoropyrimidines shift the equilibrium towards the RNA-like C3′endo form due to a higher electronegativity [10] (Figure 2B). A comparison of 2′-fluoro and 2′-amino selected aptamers targeting the keratinocyte growth factor showed that the 2′-fluoro aptamers possessed superior binding affinity to the 2′-amino modified aptamer [29]. Indeed, the 2′-amino-pyrimidines appear to have fallen out of favour for the development of therapeutic aptamers [30].

The alkyl derivatives are possibly the most studied 2′-modification, with the 2′-*O*-methyl modification used quite extensively to modify aptamers. There are two main reasons for the incorporation of this modification into the aptamers: firstly, the 2′-*O*-methyl nucleotides are far cheaper to synthesise than the 2′-fluoro or 2′-amino-pyrimidines; and secondly, the 2′-*O*-methyl bases are naturally occurring nuclease resistant nucleotides [30]. As with the 2′-fluoro modification, the 2′-OMe modifications adopt a C3′-endo conformation [34]. The first aptamer (Macugen^®^, pegaptanib sodium, Valenat Pharmaceuticals) approved by the US Food and Drug Administration (FDA) in 2004 targets vascular endothelial growth factor for the treatment of age-related macular degeneration. This aptamer was modified with 2′-fluoro-pyrimidines and 2′-*O*-methyl-purines. Together with a 3′-cap and a polyethylene glycol molecule, the half-life of Macugen^®^ was extended to 131 h [35,36].

Locked nucleic acids (LNAs) were originally developed by Wengel and Imanishi in 1998 [37,38]. In this case the 2′-oxygen has been linked to the 4′-carbon of the ribose by a methylene bridge, thus completely locking the sugar in to a 3′-endo conformation [39] (Figure 2). The introduction of LNAs into the nucleic acid sequence has been shown to increase the binding affinity, thermodynamic stability and prevent serum degradation of oligonucleotides (Table 2). There are several forms of LNAs that have been developed—the β-D-ribo isomer, the α-L-xylo-LNA isomer, the β-D-xylo LNA isomer and the α-D-ribo isomer—all with similar thermostability profiles [40]. Based on the furanose conformation of LNAs, it would appear to be possible to directly substitute 2′fluoro-pyrimidines with LNAs during post-SELEX modification processes. The 2′-OMe and 2′-F-RNA bases confer a C3′endo/north conformation, similar to that seen with LNAs, though there is a restriction in the pseudo-rotation due to the locking bridge in the LNA structure. However, studies whereby nucleotides have been replaced with LNAs have shown a complete loss of binding affinity, depending on the positioning of the LNA within the aptamer [31].

**Figure 3.**
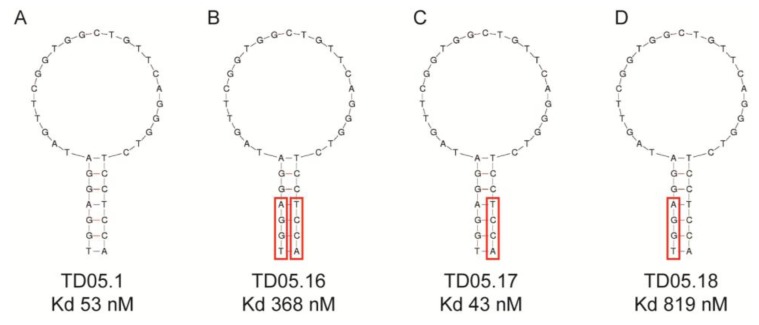
The insertion of modified bases into the structure of an aptamer can have profound effects on binding affinity. The DNA aptamer TD.05 was modified with locked nucleic acids in the stem region. (**A**) unmodified DNA aptamer; (**B**) the last four bases of the aptamer in the stem region were replaced with locked nucleic acids; (**C**) only the last four bases on the 3′-end of the aptamer were replaced with locked nucleic acids; (**D**) the first four bases on the 5′-end were modified with locked nucleic acids. Red boxes indicated substituted bases. Modified from [43].

This study replaced consecutive bases with LNAs and thus may have produced a distinctly different conformational shape from the original aptamer. Recent studies suggest that replacing every third nucleotide with LNAs would yield a near-canonical A-form heteroduplex, similar to natural RNA strands [41]. Again, any modification of the aptamer, especially in the binding region could lead to a complete loss of binding capacity as seen with the thrombin binding DNA aptamer. A single LNA was inserted into the aptamer at the 3′-end of the sequence resulting in a decreased binding affinity. Other attempts to modify this aptamer failed to improve binding affinity though 3′-capping of a different thrombin aptamer with an LNA had little effect on binding [41]. These results suggest that while it may be possible to predict to some extent the effect that modifications may have on binding affinity, it is still a trial and error process, especially in the case of substituting DNA nucleotides for LNAs. However, if there is a distinct binding region in the aptamer structure, such as that seen with the Sgc8 T cell leukaemia aptamer or the TD05.17 membrane bound IgM B cell receptor aptamer produced by the Tan group [32,42], it should theoretically be possible to modify the DNA aptamers in the non-binding stem region of the aptamer without a loss in binding affinity [41,43] (Figure 3). However, as Figure 3 shows, replacing purines or pyrimidines in the stem region with LNAs may affect the binding affinity or the aptamer. In this case it appears that the substitution of LNAs in the 5′-end of the aptamer (Figure 3B,D) had a detrimental effect on binding affinity, probably due to conformational changes which affected the binding region. However, substitutions in the 3′-end of the aptamer slightly increased binding affinity (Figure 3C). This study shows that even the stem region can be involved in binding to the target.

In comparison with LNAs, the unlocked nucleic acid (UNA) is an acyclic ribose derivative that has increased flexibility. UNAs lack the C2′-C3′ bond which confers the flexibility observed in this modified nucleotide [44] (Figure 2A). Whereas LNAs can increase the melting temperature of the nucleotide by 1 °C–10 °C per LNA insertion, UNAs will typically reduce the melting temperature by up to 5 °C–10 °C, though they still retain their resistance to nucleases [41,44]. As with LNAs, depending on the positioning of the UNA into the aptamer structure, these UNAs can either decrease or increase the specificity of target binding. UNAs are much easier to synthesise and produce higher yields than LNAs [45]. Interestingly, while LNA modification of the thrombin binding aptamer resulted in lower binding affinity, the insertion of UNAs into the sequence actually increased the binding affinity [41,44].

## 3. Modified-SELEX and the Incorporation of Serum-Stable Nucleotides

Post-SELEX modification can adversely affect the binding affinity and abolish specificity in target interaction. This has had a limiting effect on the use of aptamers as therapeutic agents. Therefore, being able to incorporate modified nucleic acids into the RNA transcription reaction that forms part of each iterative round of selection greatly increases the chance of successfully selecting RNA aptamers with effective serum stability. A key aspect to this has been the demonstration of using modified nucleotides as substrates of the transcription enzymes [46]. The discovery and production of enzymes that are capable of an *in vitro* transcription reaction incorporating modified bases into the nucleic acid strands has revolutionised the use of aptamers, and nucleic acids in general, as therapeutic agents. Seminal work by Padilla and Sousa demonstrated that the Y639F variant of T7 RNA polymerase could incorporate 2′-amino and 2′-fluoro-pyrimidines into the transcription reaction [47]. RNA polymerases, such as T7 RNA polymerase and AMV reverse transcriptase have been shown to be capable of incorporating modified bases during the transcription process [26,46]. To date, 2′-aminopyrimidines, 2′-*O*-methylpurines, and 2′-fluoropyrimidines have been incorporated in this way [46,48,49].

One of the reasons that RNA aptamers have been consistently used for *in vivo* applications is its ability to incorporate modified nucleotides into the selection process during the transcription reaction. In contrast, DNA polymerase chemistry has lagged behind the ability to use RNA polymerases such as T7 and SP6. There are now several commercially available thermostable DNA polymerases capable of inserting modified nucleotides during the PCR reaction. It has been suggested that DNA polymerases derived from *Thermococcus kodakaraensis*, such as *KOD Dash*, are the most efficient tools for incorporation of modified DNA [29]. Modifications or substitutions at the C5 position of pyrimidines or the C7 position of 7-dezapurines are fairly well tolerated by DNA polymerases [50]. Indeed, the *KOD Dash* polymerase has successfully been used to incorporate a modified thymidine analogue with a cationic ammonium group attached via a hydrophobic hexamethylene linker at the C5 position during the selection process [51]. As well, a recent report has shown the incorporation of uracil bases modified at the C5 position with a *N*-(2-(*N*^6^-adeninyl)ethyl))carbamylvinyl group during selection. When compared to natural aptamers selected against the same target, the modified DNA aptamer showed substantially higher binding affinities (1.1 μM vs 0.039 and 0.086 μM) [50]. Of note, Vaught and colleagues have successfully incorporated six different C5-modified dUTP derivatives using KOD Dash or Deep vent polymerases during PCR and were able to generate DNA aptamers against a previously refractory protein, tumour necrosis factor receptor superfamily member 9 [52]. It was this work that led to the generation of SOMAmers, or slow off-rate modified aptamers, which can be used to select against any target, even previously difficult protein targets for which standard RNA and DNA SELEX proved to be incapable of producing high affinity binding species [53]. This is due to the fact that these modified nucleotides increase the structural diversity, thus broadening the range of accessible protein targets [54].

Interestingly, KOD, Phusion High Fidelity DNA polymerase, Superscript III and the T7 polymerase have all been shown to be capable of incorporating LNA triphosphates into DNA and RNA strands [41,45,55,56], which opens up a new era in aptamer selection strategies. However, lower coupling efficiencies may preclude classical SELEX which involves 8–12 iterative rounds. Indeed, a recent study using a DNA random library and High Fusion DNA polymerase and KOD XL DNA polymerase successfully incorporated LNA-ATP into the DNA strand of 40 nucleotides. However, the level of LNA ATP incorporation dropped from 20.5% in round one to 6.6% in round 7. This decrease was more pronounced when using LNA-TTP, with a drop from 31% at round one to 6.8% at round 3 [57]. Using capillary electrophoresis SELEX, LNA aptamers were selected against thrombin using a mix of KOD polymerases, though there was no discussion of incorporation efficiency [58]. The authors did note, however, that selection was not simple [59]. Further work by this group used LNAs in the primer region of the randomised library for selection, a strategy that proved successful for the *in vitro* selection of LNA aptamers [59,60]. While these results have shown that it is possible to insert LNAs into the DNA aptamers during the selection process, additional modifications have been required to improve the reaction efficiency before DNA aptamers can compete with their RNA counterparts in traditional SELEX selection. Pinheiro and colleagues developed new Tgo DNA polymerase variants which were capable of incorporating xeno-nucleic acids during transcription and reverse transcription [61].

## 4. Functionalisation as A Means of Producing Effective Diagnostic Tools

A recent article in this journal has reviewed the use of aptamers as efficient tools in numerous analytical applications [62]. These analytical applications do not necessarily require a serum stable aptamer though they would certainly be advantageous. Diagnostic applications requiring high serum stability are those with future *in vivo* applications. The ability to attach any reporter molecule to the aptamer structure has far reaching implications in the field of cancer medicine. The ability to detect small cancer foci and monitor disease response to treatment has the potential to guide and influence clinicians decision-making with regard to therapeutic choices. Additionally, if these molecules could both act as imaging agents and therapeutic tools at the same time, there would be long-term benefits to the patient. Dyes, such as Cy5 [63], have been superseded by the advent of quantum dots. There have been a number of papers published over the last decade describing the use of quantum dots (QDs) as novel *in vivo* imaging agents [64–67]. QDs are small novel fluorophores that have distinct emission profiles based on their sizes. One single QD can have multiple copies of the same aptamer or different aptamers bound to their surface [68,69]. Each aptamer can also be base-paired to a complimentary strand that carries a quencher, with this strand being displaced upon binding to its target, thus resulting in a large increase in fluorescence emission [70]. However, other quenchers can also be used for *in vivo* applications, of which doxorubicin (Dox) is very promising in cancer medicine. Dox is a commonly used frontline anthracycline chemotherapeutic drug that intercalates into double-stranded GC sequences of DNA and RNA. The use of Dox is limited in dosage due to cardiotoxicity. Through the use of targeted therapeutics, the dose of the drug can be reduced, thus allowing either a more prolonged exposure of the drug to cancer cells and/or repeated dosing. Dox has known fluorescent properties which are rapidly quenched upon intercalation into DNA [71,72]. Through a Bi-Fluorescence Resonance Energy Transfer (FRET) mechanism, the QDs and Dox act as donor-acceptor, where the fluorescence of the QDs is quenched by the Dox absorbance, and the Dox fluorescence is quenched by the aptamer in a donor-quencher manner. When this 2′-fluoropyrimidine modified aptamer-Dox-QD is delivered *in vivo* no fluorescent signal is observed. However, once the aptamer has specifically internalised into the cell, there is a gradual release of Dox from the aptamer which then “switches on” the fluorescence of the QD, thus allowing visualisation of the cancer cells [73]. This same aptamer has also been thermally cross-linked to superparamagnetic iron oxide nanoparticles (TCL-SPION), which can be used as magnetic resonance imaging (MRI) contrast agents. With the addition of Dox to the aptamer stem, this nanoparticle has been shown to effectively target prostate cancer cells [74]. Thus, this study indicates the potential of aptamers to act as theranostic agents. Indeed, as simple as Dox is to intercalate into the stem of aptamers, so it is a relatively straightforward procedure to link siRNA to the end of aptamers [75]. As well, simple fluorophores can be attached to the end of the aptamer and the siRNA sequence, thus allowing real time *in vivo* localisation studies. This is an important consideration with siRNA delivery to ensure that the siRNA remains linked to the aptamer until specific delivery to the correct cell.

## 5. Conclusions

With the advances in nucleic acid chemistry and technological applications, it is now possible to use aptamers in almost every application that antibodies have been successfully used for to date. Unlike antibodies that may lose their specificity or sensitivity through functionalisation, aptamers generally do not. As well, as aptamers are far smaller in size than antibodies and even antibody fragments, the addition of drugs or fluorescent molecules will not severely impact on their ability to penetrate deeply into organs or tumours. With the ability of modifying these nucleic acids through the selection process, and using rational design to modify them post-SELEX, aptamers have the ability to be used successfully *in vivo*. The ability to engineer these aptamers to be nuclease resistance through simple nucleotide swaps and functionalisation will revolutionise the medical field. With one aptamer already approved by the FDA, and numerous more in clinical trials, it will only be a short time before these novel small molecules enter the mainstream pharmaceutical pipelines.

## Figures and Tables

**Table 1.** *In vitro* stability of modified aptamers.

**Modification**	**Aptamer**	**Half-Life**	**Reference**
Unmodified RNA	Trypanosome	<1 s	[10]
2′-FP	TTA1	42 h	[31] out of order
2′OMe	TTA1	49 h	[31] out of order
LNA	TTA1	53–72 h	[31]
2′FP & 2′NH_2_	Trypanosome	36 h	[10]
2′FP	Trypanosome	81 h	[10]
2′NH_2_	Trypanosome	367 h	[10]
LNA	sgc8c (c8L3)	3 h	[32]out of order
2′FP	RET Receptor Tyrosine Kinase	6 h	[33]out of order

*Notes: Abbreviations*: 2′FP: 2′-Fluoropyrimidines; 2′OMe: 2′*O*-methyl: LNA: Locked nucleic acid; 2′NH_2_: 2′-aminopyrimidine.

**Table 2.** Incorporation of locked nucleic acids (LNA) can improve serum stability [41].

**Modification**	**Half-Life**
2 × LNA	2.5 fold
3 × LNA	11 fold
8 × LNA	15 fold
